# Multi‐omics analysis of pyroptosis‐related genes for prognosis and immune landscape in head and neck cancer

**DOI:** 10.1002/ctm2.70144

**Published:** 2024-12-17

**Authors:** Shikang Zheng, Qinghua Liu, Cheng Wang, Rongqi Zhang, Xin Peng, Junda Fan, Haiming Xu, Xiazhi Pan, Nanxiang Chen, Mingbo Liu, Kai Zhao

**Affiliations:** ^1^ Department of Otolaryngology Head and Neck Surgery Hainan Hospital of Chinese PLA General Hospital Sanya China; ^2^ Medical School of Chinese PLA, Haidian Beijing China; ^3^ Beijing Institute of Technology Beijing China; ^4^ School of Clinical Medicine Shandong Second Medical University Weifang China; ^5^ Department of Otolaryngology Head and Neck Surgery the Sixth Medical Center of Chinese PLA General Hospital Beijing China; ^6^ National Clinical Research Center for Otolaryngologic Diseases Chinese PLA General Hospital Beijing China

1

Dear Editor,

Despite the demonstrated efficacy of immunotherapy in various cancers, treating head and neck squamous cell carcinoma (HNSCC) continues to pose significant challenges.^1^, ^2^ Pyroptosis, a distinct form of programmed cell death, is intricately associated with tumour progression and immune response modulation.^3^, ^4^ This study undertakes a comprehensive multi‐omics analysis to elucidate the complex role of pyroptosis‐related genes (PRGs) in the context of HNSCC, with the objective of developing a robust prognostic signature that could substantially advance the understanding of the prognosis of HNSCC and its associated immune landscape.

Figure 1A provides a comprehensive overview of the study's workflow, delineating the principal steps and methodologies employed in our investigation. The study encompasses 528 cancer samples and 44 normal controls from the TCGA database, along with 270 cancer samples from the GEO database. We identified 64 PRGs, of which 51 were differentially expressed in HNSCC tissues (Figure S1A). Survival analysis showed that 33 of these genes were linked to patient outcomes (Figure S2). A prognostic network was developed to elucidate the interrelationships among these genes (Figure 1B). Analysis revealed that 409 of 510 samples had PRG mutations, an 80.2% mutation rate (Figure S1B). Additionally, PRGs often showed copy number variations (CNVs), with gains or losses illustrated in Figure S1C, and their chromosomal distribution was shown in Figure S1D.

**FIGURE 1:**
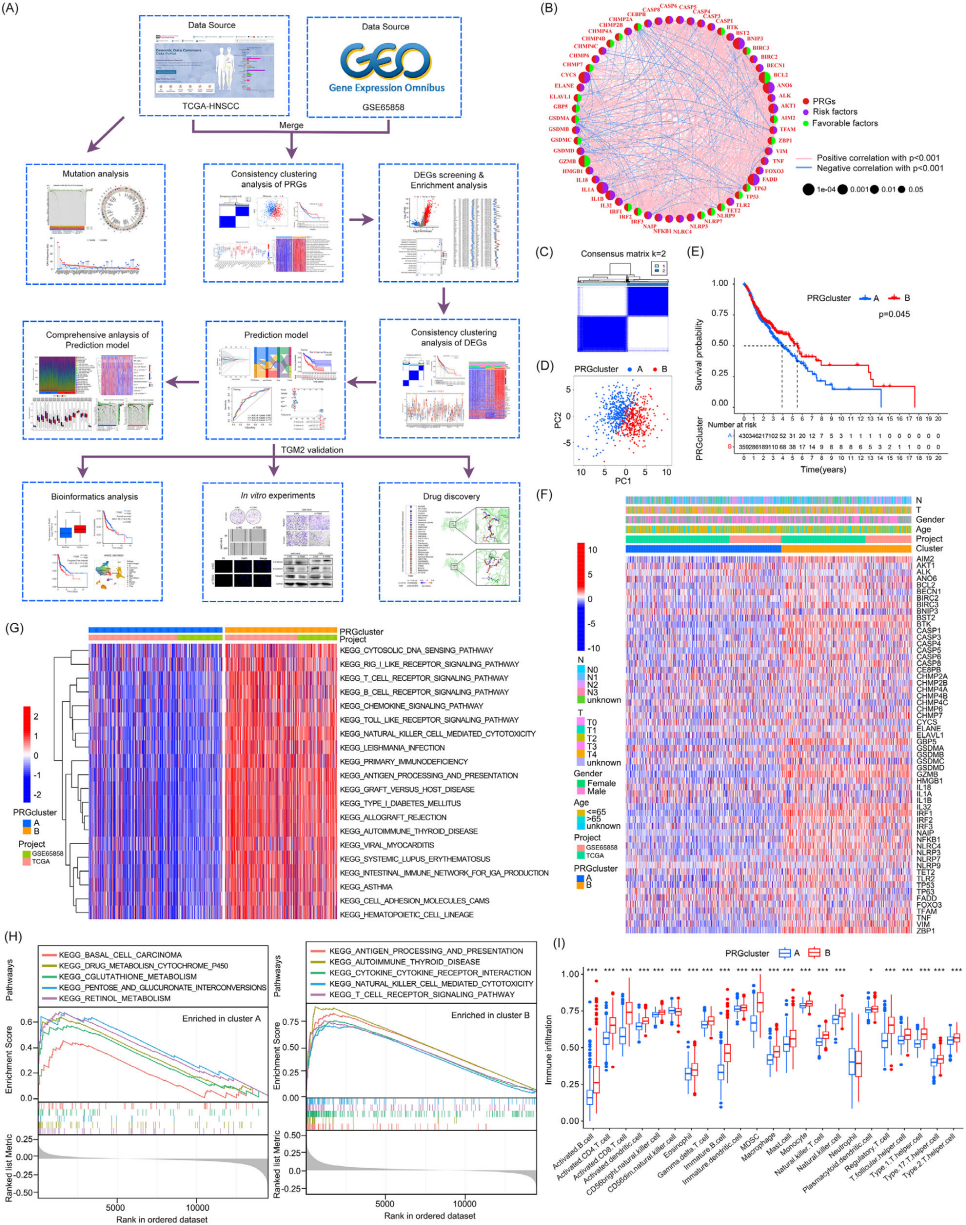
Identification of pyroptosis‐related genes (PRGs)‐related clusters in head and neck squamous cell carcinoma (HNSCC). (A) The entire workflow of the research. (B) Correlation network of prognostic PRGs. (C) The consensus matrix heatmap of PRGs. (D) Principal Component Analysis (PCA) demonstrates good discrimination between the two subgroups. (E) Kaplan–Meier curve analysis of survival differences among the two subgroups. (F) Heatmap illustrates the PRGs and clinical features between different clusters. (G) Pathways enriched by Gene Set Variation Analysis (GSVA) in different pyroptosis subtypes. (H) Pathways enriched in clusters A or B according to Gene Set Enrichment Analysis (GSEA) analysis. (I) Box diagram of immune infiltration between the various subtypes.

Hierarchical clustering analysis identified two clusters in HNSCC (Figure 1C,D), with cluster B showing a significantly better prognosis than cluster A (Figure 1E). The clinical characteristics and PRGs expression profiles associated with these subtypes are presented in Figure 1F. Gene Set Variation Analysis (GSVA) and Gene Set Enrichment Analysis (GSEA) analyses demonstrated that cluster B was associated with immune‐related pathways, while cluster A was enriched in metabolic pathways (Figure 1G,H). This observation is further corroborated by ssGSEA, which revealed a higher degree of immune cell infiltration within cluster B (Figure 1I).

We further identified 717 differentially expressed genes (DEGs) related to pyroptosis subtypes (Figure 2A), with 169 DEGs significantly affecting prognosis (Figure S3A). The results of the enrichment analysis for the DEGs were presented in Figure S3B. We further performed a clustering analysis and found that *k* = 3 was optimal (Figure 2B,C). Notably, patients in group C had a better prognosis than those in other groups (Figure 2D). Moreover, there is a notable overlap in clinical traits and DEG expression between geneCluster group C and PRGCluster cluster B (Figure 2E). To develop a novel prognostic signature for HNSCC, randomly selected patients were assigned to a training cohort for signature development and a validation cohort for evaluation. Through the application of Least Absolute Shrinkage and Selection Operator (LASSO) regression and multivariate Cox regression analyses, seven key DEGs were identified as essential for the construction of the prognostic signature (Figure 2F,G and Table S1). The signature's gene expression, risk score differentiation, prognoses for high‐ and low‐risk groups and prediction accuracy were consistent across the training group (Figure 2H–K), validation group (Figure 2L–O) and the overall cohort (Figure 2P–S). Both univariate and multivariate analyses, along with the concordance index (*C*‐index) curves, substantiated that the prognostic signature provided superior predictive effect for the survival of HNSCC patients compared to other clinical characteristics (Figure 2T,U). A nomogram was developed to estimate survival rates across different follow‐up periods, utilising clinical features and risk scores (Figure 2V). The calibration curve revealed an excellent agreement between the survival probabilities forecasted by the nomogram and the actual patient outcomes, signifying a high level of predictive precision (Figure 2W). Additionally, cluster B in PRGCluster and group C in geneCluster were linked to better prognoses and lower risk scores (Figure S4A–C), which also exhibited higher expression of PRGs (Figure S4D,E).

**FIGURE 2:**
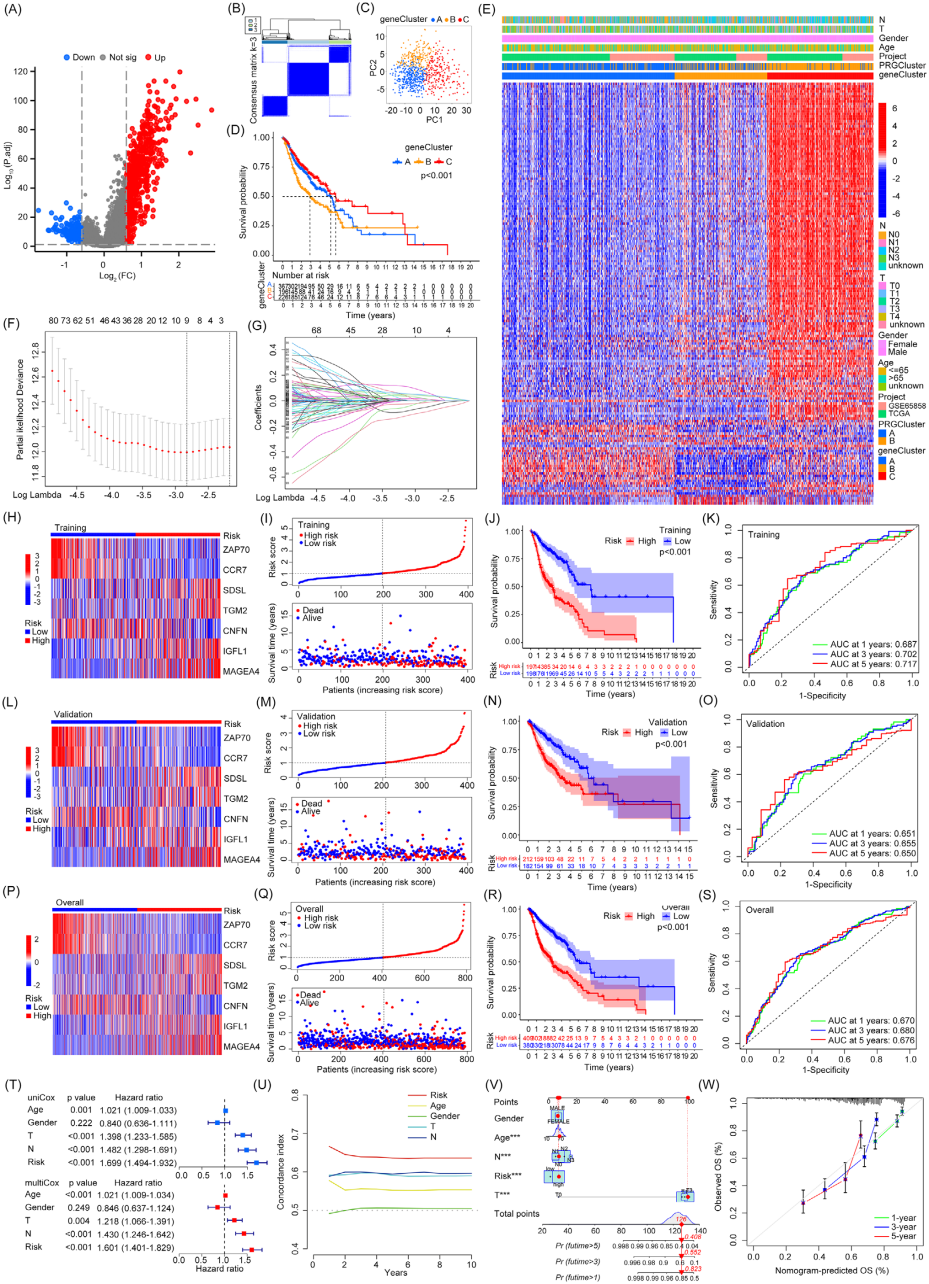
Construction and validation of prognostic signature based on pyroptosis‐related genes (PRGs)‐associated differentially expressed genes (DEGs) in head and neck squamous cell carcinoma (HNSCC). (A) Volcano plot of DEGs between PRGs‐related clusters. (B) The consensus matrix heatmap of DEGs. (C) Principal Component Analysis (PCA) analysis between the three subgroups. (D) Kaplan–Meier curve analysis of survival differences among the three subgroups. (E) Heatmap illustrates the DEGs and clinical features between different clusters. (F) Cross‐validation in the LASSO algorithm. (G) The LASSO coefficient plot of DEGs. (H–K) Heatmap of signature genes (H), distribution of risk groups and its relationship with survival status and time (I), Kaplan–Meier curves of overall survival of high‐ or low‐risk patients (J) and receiver operating characteristic (ROC) curve of risk score (K) in training group. (L–O) Heatmap of signature genes (L), distribution of risk groups and its relationship with survival status and time (M), Kaplan–Meier curves of overall survival of high‐ or low‐risk patients (N) and ROC curve of risk score (O) in validation group. (P–S) Heatmap of signature genes (P), distribution of risk groups and its relationship with survival status and time (Q), Kaplan–Meier curves of overall survival of high‐ or low‐risk patients (R) and ROC curve of risk score (S) in overall cohort. (T) Univariate/multivariate Cox regression analysis of clinical features and risk score in HNSCC patients. (U) The *C*‐index curve of the prognostic signature and clinical characteristics. (V) The nomogram established by prognostic signature and clinical characteristics. (W) Calibration curves for nomogram validation.

Immune cell infiltration analysis identified significant associations between immune cells, risk scores and signature genes (Figures 3A–C and S5). The high‐risk group exhibited reduced immune function and lower expression of immune checkpoint genes, suggesting greater immune evasion and poor response to immunotherapy (Figure 3D,E). Tumour microenvironment (TME) scores,^5^ tumour immune dysfunction and exclusion (TIDE) scores^6^ and immunophenoscore (IPS)^7^ collectively indicated that the low‐risk group had better immune infiltration and responses, while the high‐risk group showed greater immune evasion, potentially reducing its response to immune checkpoint blockade (ICB; Figure 3F–H). As well as gene mutation frequency, we assessed tumour mutational burden (TMB) between high‐ and low‐risk groups. Our results indicated that the TP53 gene showed a high mutation frequency in both groups (Figure 3I). Furthermore, the high‐risk cohort exhibited an increased TMB level, indicating a potential link between elevated TMB and heightened genomic instability, which may be associated with a less favourable prognosis (Figure 3J,K).

**FIGURE 3:**
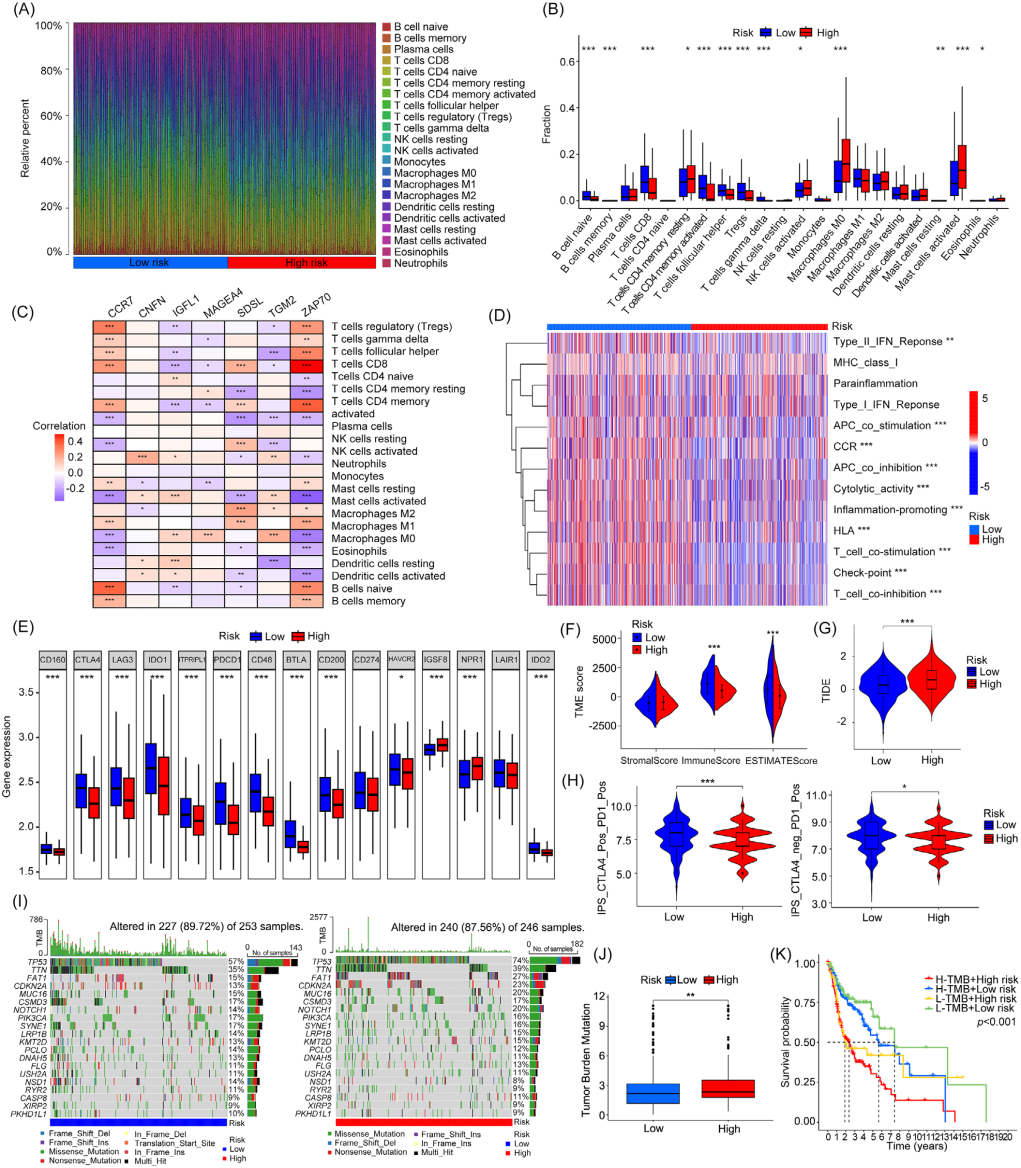
Comprehensive analysis of prognostic signature‐related immune and genetic mutational landscapes. (A) Bar chart of immune cell percentages between high‐ and low‐risk groups. (B) Box diagram of immune infiltration fraction between high‐ and low‐risk groups. (C) Heatmap of the correlation between signature genes and infiltrated immune cells. (D) Disparity of immune function between high‐ and low‐risk groups. (E) Variation of immune checkpoints genes expression between high‐ and low‐risk groups. (F–H) Tumour microenvironment (TME) scores (F), Tumour immune dysfunction and exclusion (TIDE) scores (G) and Immunophenoscore (IPS) (H) of high‐ and low‐risk groups. (I) Distribution of the high‐frequency mutated genes in the high‐ and low‐risk groups. (J) Tumour mutational burden (TMB) differences between high‐ and low‐risk groups. (K) The Kaplan–Meier survival curves of patients stratified by TMB and risk scores.

Among the candidate genes, Transglutaminase (TGM2) was selected for experimental validation due to its uncharacterised role in HNSCC. Our observations revealed a significant upregulation of TGM2 in HNSCC tissues, which was associated with adverse prognostic outcomes (Figure 4A,B). Additionally, single‐cell RNA sequencing data^8^ indicated that TGM2 is predominantly expressed in mast cells and monocytes/macrophages within HNSCC (Figure S7). To substantiate these findings, we employed TGM2‐siRNAs to achieve TGM2 knockdown, and the efficiency of the siRNAs was verified (Figure 4C). The suppression of TGM2 expression markedly reduced the proliferation (Figure 4D–F), migration and invasion abilities of HNSCC cells (Figure 4G–J), while promoting cell death (Figure 4K,L) and inhibiting epithelial–mesenchymal transition (EMT; Figure 4M), thereby elucidating the oncogenic function of TGM2. Furthermore, utilising the GSCA platform,^9^ we conducted an analysis to explore the correlation between Genomics of Drug Sensitivity in Cancer (GDSC) pharmacological agents and TGM2 mRNA levels (Figure 4N). Subsequently, a Venn analysis was conducted, integrating these data with drug sensitivity information derived from the risk signature (Figure S6) and TGM (Figure S8), which led to the identification of two potential therapeutic agents: Dasatinib and WH‐4‐023 (Figure 4O). Finally, AutoDocktools was employed to examine the docking interactions between TGM2 and these two compounds (Figure 4P).

**FIGURE 4:**
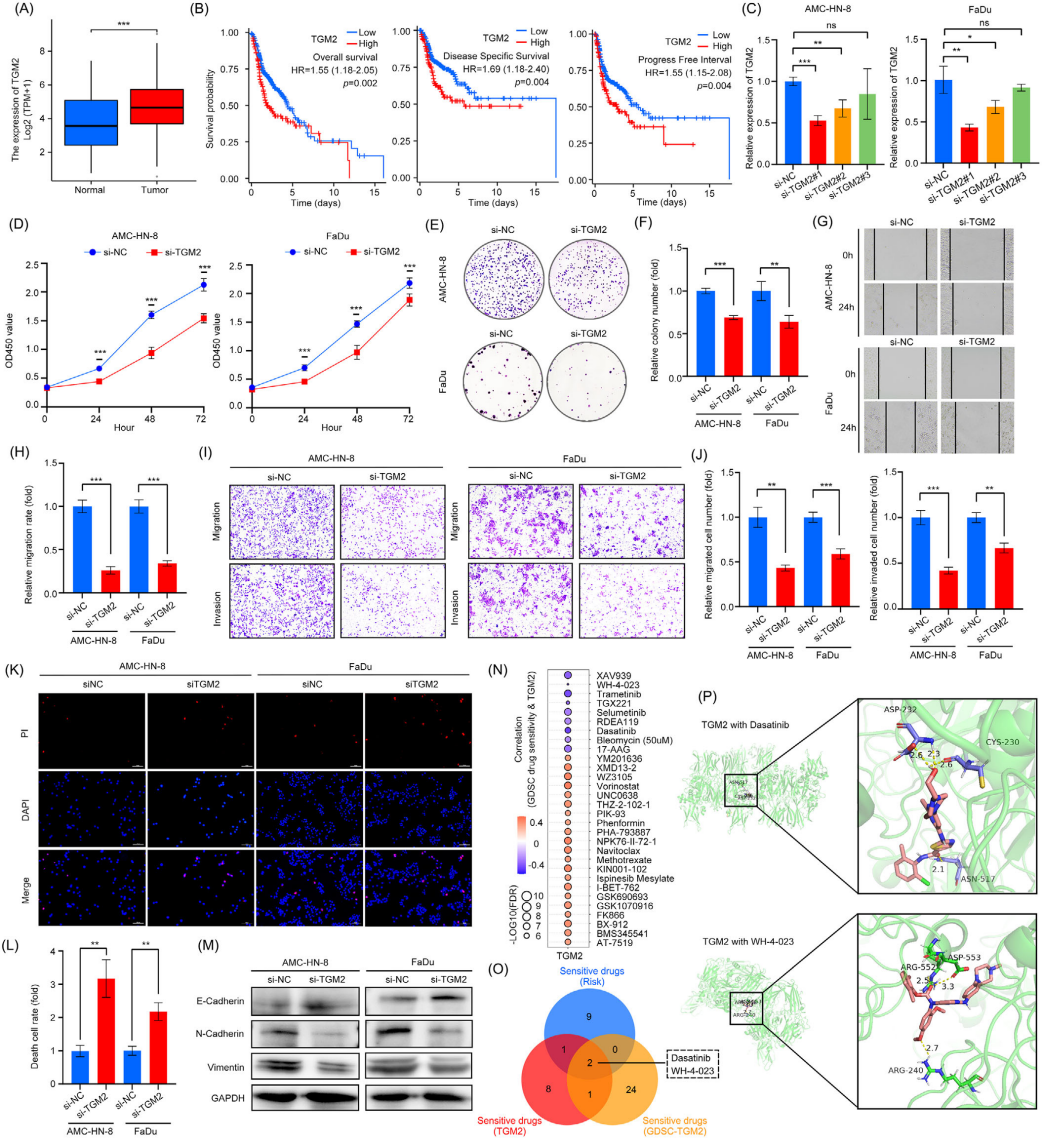
Functional validation of TGM2 and chemotherapy drug sensitivity evaluation. (A) TGM2 expression in normal and head and neck squamous cell carcinoma (HNSCC) tissues. (B) Kaplan–Meier curve analysis of survival differences among the patients with diverse TGM2 expression. (C) Real‐time polymerase chain reaction (qPCR) analysis of TGM2 knockdown validation in HNSCC cells. (D) CCK8 assays of cell proliferation in TGM2 silenced HNSCC cells. (E, F) Colony formation assays (E) and associated quantitative analysis (F) in TGM2 silenced HNSCC cells. (G, H) Scratch assays (G) and associated quantitative analysis (H) in TGM2 silenced HNSCC cells. (I, J) Transwell assays (I) and associated quantitative analysis (J) in TGM2 silenced HNSCC cells. (K, L) PI staining assays (K) and associated quantitative analysis (L) in TGM2 silenced HNSCC cells. (M) Western blots of epithelial–mesenchymal transition (EMT) markers in TGM2 silenced HNSCC cells. (N) Correlation between GDSC pharmacological agents and TGM2 mRNA levels. (O) Venn diagram showing the intersection of risk‐sensitive drugs and TGM2‐sensitive drugs predicted by the pRRophetic R package, and TGM2‐associated GDSC agents. (P) Presentation of molecular docking of TGM2 with Dasatinib (−7.7 kcal/mol) and WH‐4‐023 (−9.2 kcal/mol).

To conclude, the current study clarifies the significance of PRGs in the prognosis of HNSCC by developing a prognostic signature that may improve the prediction of patient survival and identifying TGM2 as a potential therapeutic target, thereby providing insights into the immune landscape of HNSCC. We believe that these findings have significant practical implications for enhancing patient management and informing the development of novel therapeutic strategies for HNSCC.

## AUTHOR CONTRIBUTIONS

Shikang Zheng conducted the bioinformatic analysis and drafted the original manuscript. Qinghua Liu and Cheng Wang made significant contributions to experimental operation and data acquisition. Rongqi Zhang and Xin Peng were responsible for resources and supervision. Junda Fan and Haiming Xu handled software and visualisation. Xiazhi Pan and Nanxiang Chen were in charge of data validation and performed quality control checks. Mingbo Liu and Kai Zhao conducted the conception, funding acquisition and manuscript revision. All the authors made substantial contributions to the article and approved the final version for publication.

## CONFLICT OF INTEREST STATEMENT

The authors declare no conflicts of interest.

### ETHIC STATEMENT

The current study is based on the data available in the public databases and in vitro cell lines, ethics statement is not applicable for this study.

## Supporting information



Supporting Information

## Data Availability

The datasets used in this study can be obtained through GEO (https://www.ncbi.nlm.nih.gov/geo/), TCGA (https://portal.gdc.cancer.gov/), XENA (https://xena.ucsc.edu/), and are available from the corresponding author upon reasonable request.
