# Supplementary material for: Multi‐omics analysis of pyroptosis‐related genes for prognosis and immune landscape in head and neck cancer
Source: Clin Transl Med. 2024 Dec 17;14(12):e70144. doi: 10.1002/ctm2.70144 (PMC11652105; doi:10.1002/ctm2.70144)
Supplement: Supplementary file 1 — Supporting Information [file CTM2-14-e70144-s001.docx]

**Supporting information**

**Abbreviations**

| HNSCC | Head and Neck Squamous Cell Carcinoma |
| --- | --- |
| PRGs | Pyroptosis-Related Genes |
| LASSO | Least Absolute Shrinkage and Selection Operator |
| ROC | Receiver Operating Characteristic |
| TME | Tumor Microenvironment |
| TCGA | The Cancer Genome Atlas |
| GEO | Gene Expression Omnibus |
| GO | Gene Ontology |
| KEGG | Kyoto Encyclopedia of Genes and Genomes |
| GSVA | Gene Set Variation Analysis |
| ssGSEA | Single-sample Gene Set Enrichment Analysis |
| GSEA | Gene Set Enrichment Analysis |
| BP | Biological Process |
| CC | Cellular Component |
| MF | Molecular Function |
| DEGs | Differentially Expressed Genes |
| RS | Risk Score |
| TMB | Tumor Mutational Burden |

**Materials and methods**

**Research Strategy**

This study was designed to explore the prognostic and immunological implications of pyroptosis-related genes (PRGs) in head and neck squamous cell carcinoma (HNSCC) through a comprehensive multi-omics approach. The analysis began by identifying differentially expressed PRGs in HNSCC samples from TCGA and GEO datasets, followed by survival analysis to determine their prognostic relevance. Using consensus clustering, patients were stratified into pyroptosis-based subtypes to uncover distinct biological and immunological characteristics. To refine these findings, a secondary clustering analysis based on differentially expressed genes (DEGs) from the PRG subtypes was conducted, linking gene expression profiles to clinical outcomes. A prognostic model was then constructed using LASSO and multivariate Cox regression, incorporating key DEGs into a risk score framework for survival prediction. The model was validated across independent cohorts, and its clinical utility was further enhanced by the development of a nomogram. Finally, experimental validation and drug sensitivity analyses were performed for TGM2, a key gene identified in the study, to investigate its functional role and therapeutic potential, integrating bioinformatics predictions with in vitro assays. This integrative strategy provides a robust framework for connecting PRG activity with prognosis and therapeutic opportunities in HNSCC.

**Data Source and Processing**

Transcriptomic data, clinical data, mutation, and copy number data for HNSCC were obtained from TCGA database (https://portal.gdc.cancer.gov/). The transcriptomic data were organized and ID conversion was performed using Perl software. Searching for "HNSCC cancer survival" in the GEO database (https://www.ncbi.nlm.nih.gov/geo/), we obtained the microarray dataset GSE65858 (n=270) containing clinical information for HNSCC. The data were annotated using Perl language.

**Analysis of Differential Expression and Genomic Variations of PRGs in HNSCC**

We extracted 64 genes related to pyroptosis from previous literature reports. Using the R software "limma" package, we performed extraction and analysis of PRGs in HNSCC genomic data. The "reshape2" and "ggpubr" packages were utilized for data reshaping and visualization to generate differential expression plots for PRGs between cancer and adjacent tissues. The "maftools" package was employed for the analysis of mutation data downloaded for 409 patient samples, depicting the mutation landscape of PRGs. Furthermore, we downloaded copy number-related data for HNSCC from the UCSC Xena database (https://xena.ucsc.edu/). Using Perl language, we extracted copy numbers of cell death-related genes in the samples. The R package "RCircos" was then utilized for the chromosomal localization of copy number variations in PRGs.

**Survival analysis and subtyping of PRGs.**

We integrated gene expression information from various databases for HNSCC patients using the "limma" and "sva" packages in R, and extracted the expression profiles of PRGs. Combining this with clinical data for HNSCC patients, we utilized the "survival" and "survminer" packages for survival analysis. The "ConsensusClusterPlus" package was employed to classify distinct cohorts of HNSCC patients, followed by survival analysis based on the classification.

**Identification of Gene Pyroptosis Subtypes**

Based on the subtype results and patient clinical data, we used the "pheatmap" package to display the clinical information of patients with different subtypes. Additionally, we utilized the 'GSEABase' and 'GSVA' packages to comprehensively analyze immune cell infiltration levels and pathway differences levels across the subtypes.

**Gene Ontology (GO) and Kyoto Encyclopedia of Genes and Genomes (KEGG) Enrichment Analysis for HNSCC Cell Pyroptosis Subtypes**

Using R language, we extracted genes with significant differences from the two subtypes. Subsequently, we employed the 'clusterProfiler' package and 'org.Hs.eg.db' package for GO and KEGG pathway enrichment analysis. The filter was applied with a threshold of P < 0.05 for significance.

**Re-subtyping of differential genes**

We screened prognostically significant genes from the DEGs in pyroptosis subtypes. Subsequently, we employed the consensus clustering algorithm to these prognostically significant differential genes for further subtyping. Further analysis was conducted on the survival outcomes among different subtypes. Additionally, we performed an analysis of clinical pathological features, pathway differences, and levels of infiltration by immune cells among the subtypes.

**Development and Verification of the Predictive Risk Model**

We merged patient differential gene expression files with survival information, and Patients were randomly allocated into into two groups. Using the "glmnet" package, we performed LASSO analysis on the training set data. Employing cross-validation (cv.glmnet), we selected the optimal lambda value and built a subset. The risk score calculation formula is$\sum_{i=1}^{n} \lambda i * \beta i$, where λi denotes the expression level of each prognostic-related DEGs, and βi represents the regression coefficient (Table S1) for these genes in the model. Patients were stratified into high-risk and low-risk groups based on the median risk score.

With the assistance of the "survdiff" function and the "timeROC" function, we conducted survival difference tests for different risk groups and evaluated the model with ROC curves. Combining pyroptosis subtypes and gene subtyping results, we evaluated the risk profiles of different subtypes. Finally, we developed nomograms by integrating patient clinical and pathological factors with risk scores to analyze patient survival rates.

**Immune Cell Infiltration and Immune Correlation Analysis**

Using R language, we merged and organized data from TCGA and GEO databases into a matrix file. The CIBERSORT algorithm was then employed to estimate the proportions of different immune cell types in the samples, with CIBERSORT parameters set to perm=1000 and QN=TRUE. Based on the CIBERSORT analysis results, we selected immune cells with significantly higher relevance to risk scores for Spearman correlation tests and generated a heatmap illustrating their association.

**Mutation and Stem Cell Correlation**

We processed patient mutation data using the Perl language and plotted gene mutation waterfall charts based on the results. Additionally, we utilized the "estimate" package to assess the tumor microenvironment in different risk groups and conducted differential analysis. Finally, we downloaded pan-cancer stem cell scoring data, analyzed the relationship between tumor stem cells and RS using R language, and visualized the results with a scatter plot.

**Cell Culture and Transfection**

AMC-HN-8 and FaDu cells were sourced from Wuhan Punoase Life Technologies Co., Ltd. Transfer the cells to DMEM medium supplemented with 10% FBS and culture them at 37°C with 5% CO_2_. Cell passages were limited to 30 generations. siRNA NC and siRNA TGM2 were purchased from Guangzhou RiboBio Co., Ltd. Transfection was performed using Lipofectamine RNAiMAX reagent (Thermo Fisher Scientific). The primer sequences used in this study are included in Table S2.

**Proliferation Analysis**

CCK-8 Experiment: Cells treated with different siRNAs were seeded into 96-well plates (Corning), with two negative control wells established. After incubating for a specified period, 10 μL of CCK-8 reagent (Dojindo, Japan) was added to each well. Absorbance at 450 nm was measured with a microplate reader. (UMR9600, Hangzhou UMI Instruments CO, Ltd).

Cloning Formation Experiment: In each well of a 6-well plate (Corning), 1000 transfected cells were seeded. After 10 days, the resulting colonies were fixed and stained. Experiment results were captured using a camera, and the formed colonies were counted.

**Wound Healing and Migration/Invasion Experiments**

Wound Healing Experiments: Cultivate tumor cells in a culture dish to form a monolayer of cell population. Use a cell scraper to create a straight-line scratch on the monolayer of cells. Images were captured at 0h and 24h after creating the wound. For the migration assay, add 200 µL of the transfected cell suspension to the upper chamber of the Transwell insert (Corning). Incubate the transwell plate in the cell culture incubator for 48 hours. After incubation, photograph and count the cells that have migrated to the lower chamber. In the invasion assay, transwell inserts pre-coated with matrix gel (Corning) were used, and the rest of the procedure was the same as the migration assay. Quantitative analysis for all the above experiments was performed using ImageJ software.

**Western Blot**

Transfected HNSCC cells were lysed using lysis buffer containing 1% protease inhibitor, and protein concentration was measured using the BCA method (Thermo Fisher). Total proteins underwent 4%-12% SDS-PAGE separation, and the appropriate PVDF membrane was cut for transfer. Subsequently, the membrane was blocked using rapid blocking buffer (Beyotime). The membrane was subsequently blocked using rapid blocking buffer (Beyotime) and incubated on ice for 12 hours with specific antibodies against N-cadherin, E-cadherin, and Vimentin (Abcam UK). After washing with TBS for 30 minutes, the membrane was incubated with specific secondary antibodies for 1 hour, and signal detection was performed using a chemiluminescence kit (Thermo Fisher Science).

**Cell Death Detection**

Add 2 ml of the transfected cell suspension to the cell culture slides. When they reached a certain density, the slides were immersed in PBS for one wash, followed by fixation with tissue fixative. After fixation, the cell slides were washed with PBS to remove the fixative. Subsequently, each slide was treated with 1 milliliter of a 20 μg/ml solution of propidium iodide (PI) (Glpbio US) for 30 minutes for fluorescence staining. Following this, the fluorescence staining solution was washed off with PBS, and cell apoptosis was examined using a fluorescence microscope.

**Drug Screening**

The "pRRophetic" package was utilized for predicting drug sensitivity between the two groups, with a filtering threshold set at p < 0.001. Simultaneously, Spearman correlation coefficients between TGM2 expression levels and the sensitivity (50% inhibitory concentration, IC50) of small-molecule drugs in the GDSC database were calculated using the GSCALite online database to identify small molecules targeting TGM2. After obtaining the intersection, small-molecule drugs were selected, and their structures were downloaded from the PubChem database and processed using Chem3D software. The 3D structure of the TGM2 gene was downloaded from the PDB database (http://www.rcsb.org/). AutoDocktools software (http://vina.scripps.edu/) was used for ligand and receptor preprocessing, and Vina for execution, with pyMOL utilized for visualization.

| Genes | Risk coefficients (*βi)* |
| --- | --- |
| ZAP70 | -0.207850740714438 |
| CCR7 | -0.222914047995242 |
| SDSL | 0.253218997333069 |
| TGM2 | 0.116443201182769 |
| CNFN | -0.0641585451258766 |
| IGFL1 | 0.0812557020722391 |
| MAGEA | 0.0739536867362869 |

Table S1 Risk coefficients of seven prognosis related DEGs in signature.

| Genes | Primers | Sequence 5’-3’ |
| --- | --- | --- |
| GAPDH | Forward | GTCTCCTCTGACTTCAACAGCG |
|  | Reverse | ACCACCCTGTTGCTGTAGCCAA |
| TGM2 | Forward | ACAAATCCATCAACCGTTCC |
|  | Reverse | GCCAGTTTGTTCAGGTGGTT |

Table S2 Primers used in the research.


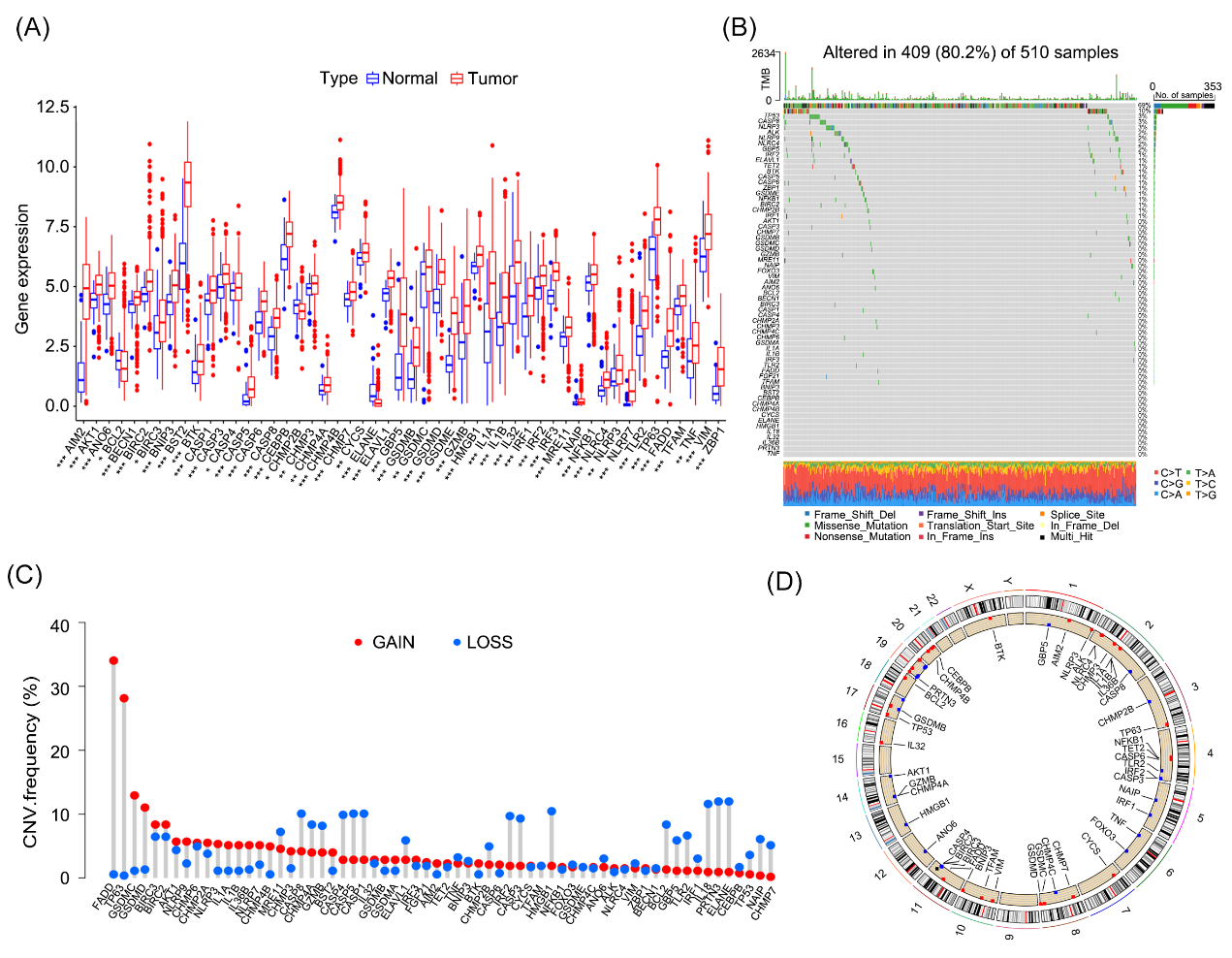


Figure S1 Differential expression and genetic alterations of PRGs in HNSCC. (A) Differential expression of PRGs between normal and HNSCC samples. (B) Mutation frequency and types of PRGs in HNSCC patients. (C) CNV status of PRGs in HNSCC. (D) Locations of CNVs of PRGs occurring on 23 pairs of chromosomes.


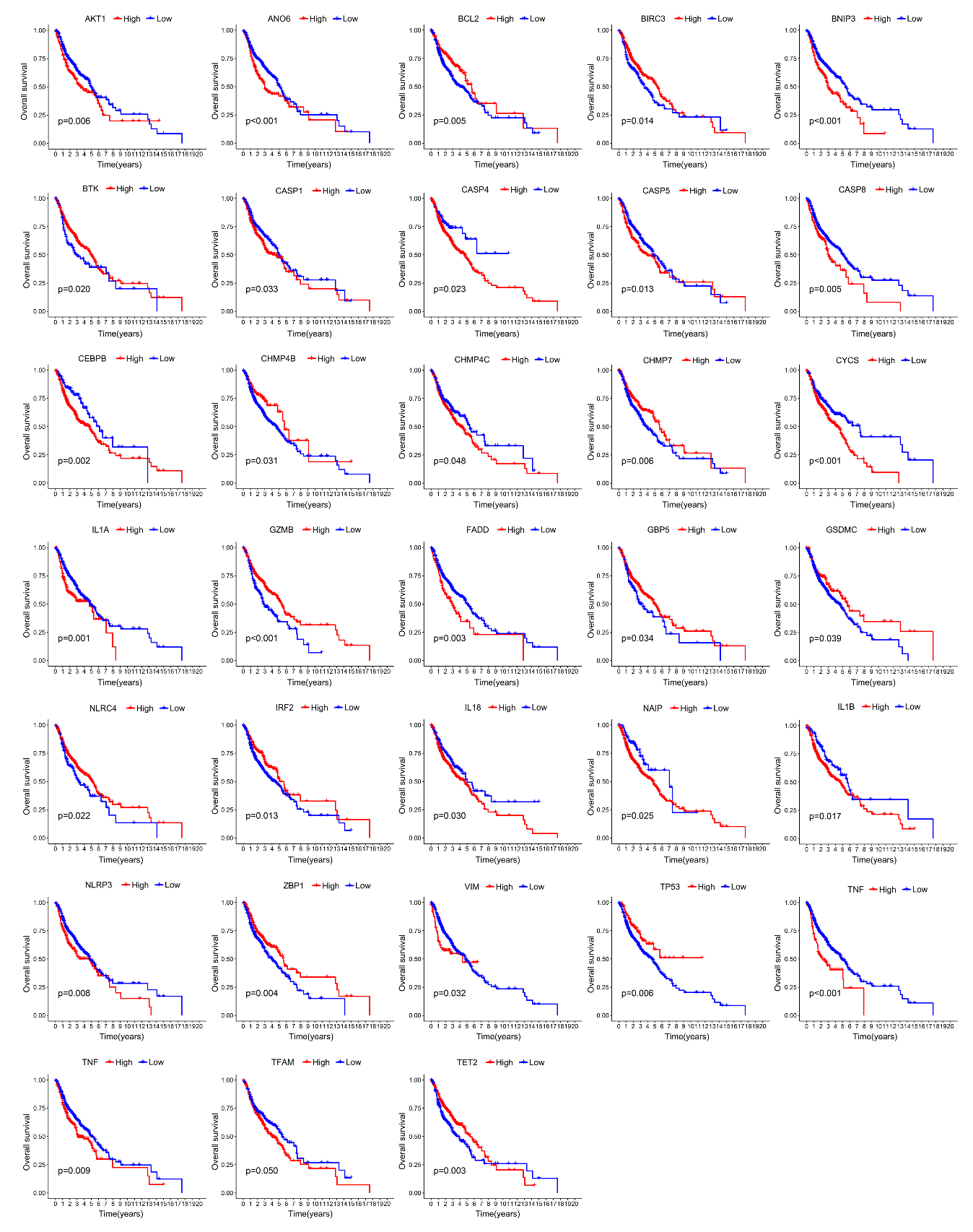


Figure S2 Kaplan–Meier curve analysis of overall survival differences among the patients with diverse PRGs expression.


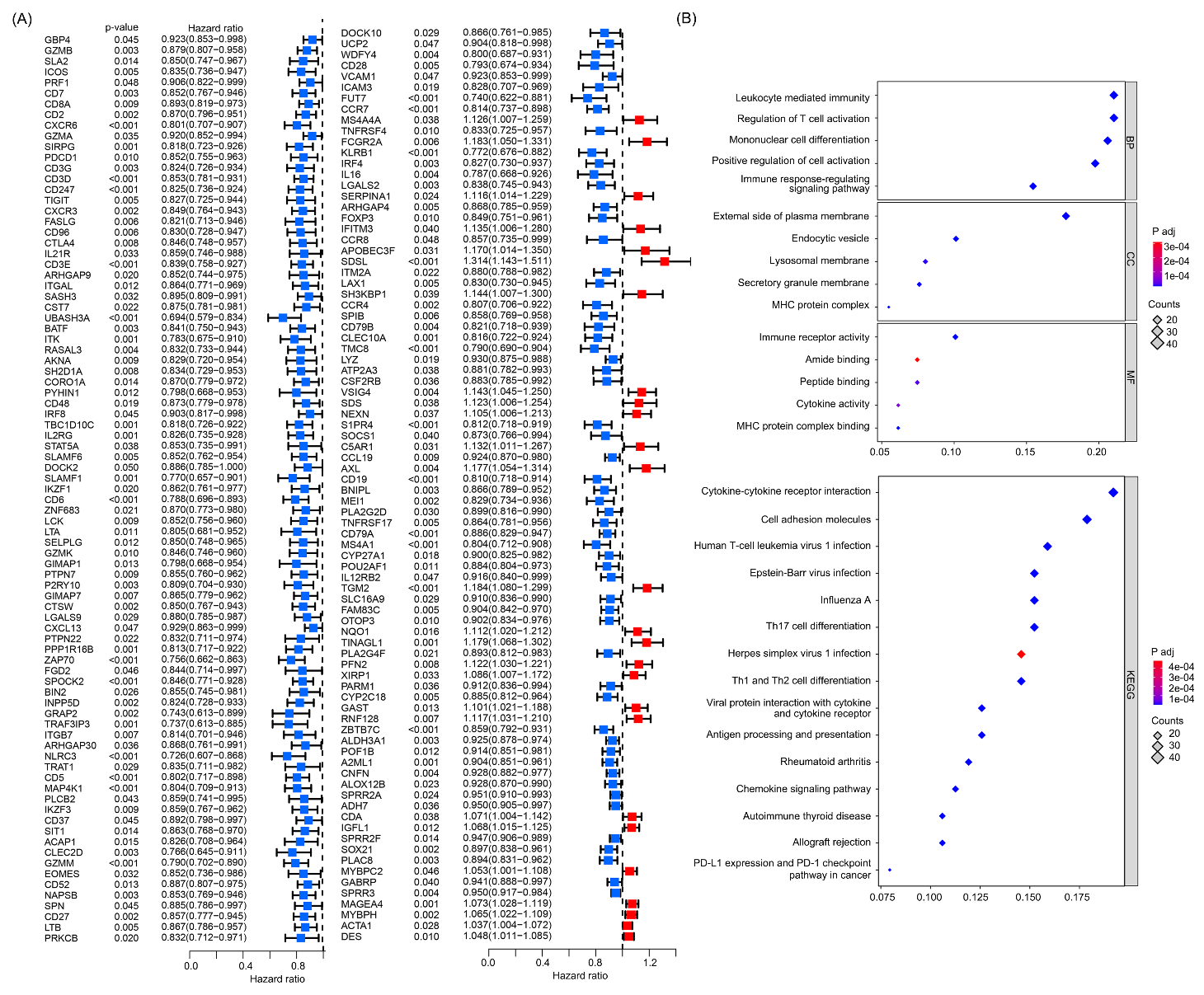


Figure S3 Prognostic and enrichment analysis of DEGs. (A) Univariate cox regression analysis of DEGs in HNSCC patients. (B) GO and KEGG enrichment analyses of DEGs.


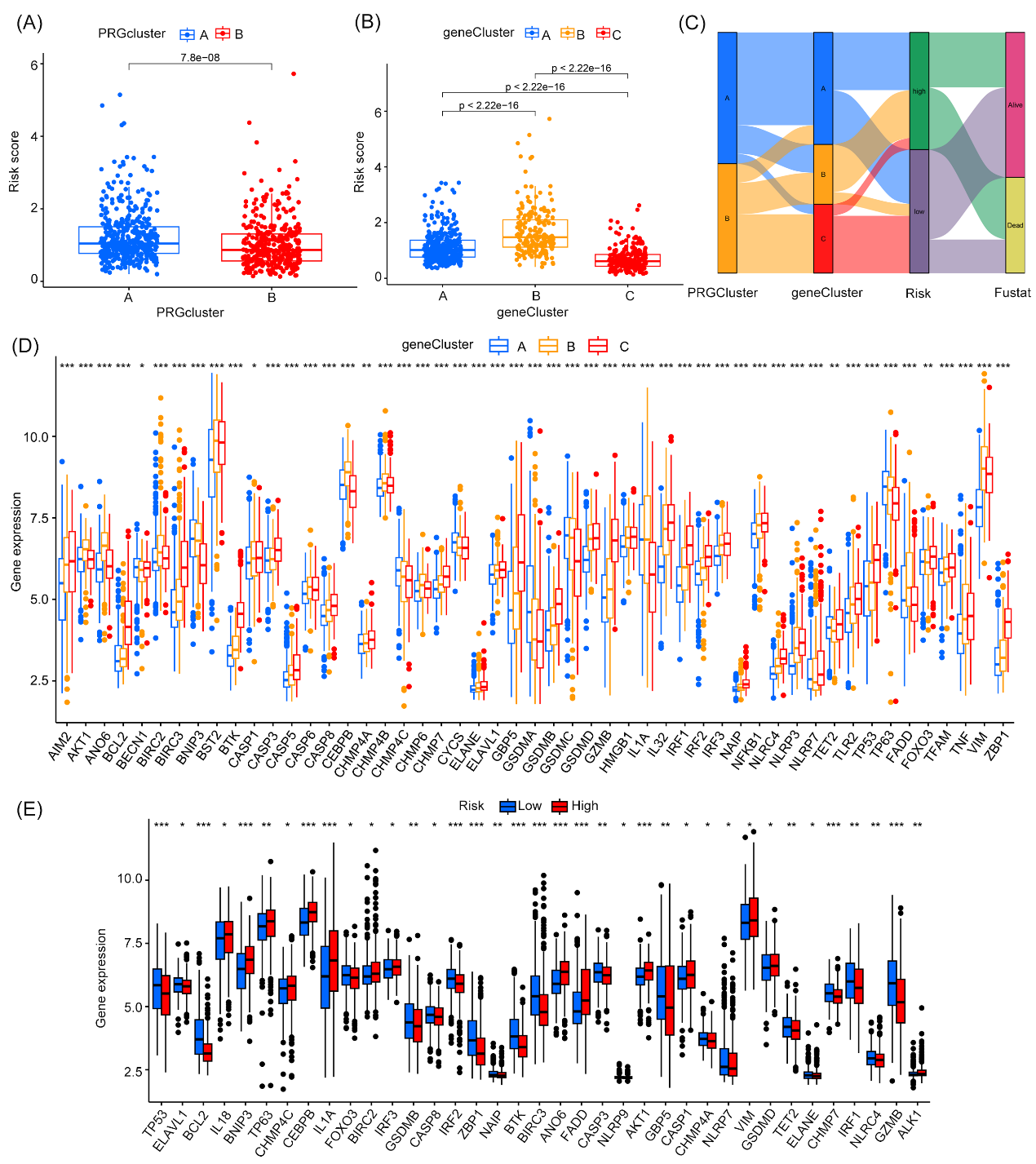


Figure S4 Correlation between gene subtyping and risk grouping. (A) Association between risk scores and PRGCluster groups. (B) Association between risk scores and geneCluster groups. (C) Sankey diagram displaying the associations between subtypes and risk groups. (D) Variation of PRGs expression between geneCluster groups. (E) Variation of PRGs expression between high- and low-risk groups.


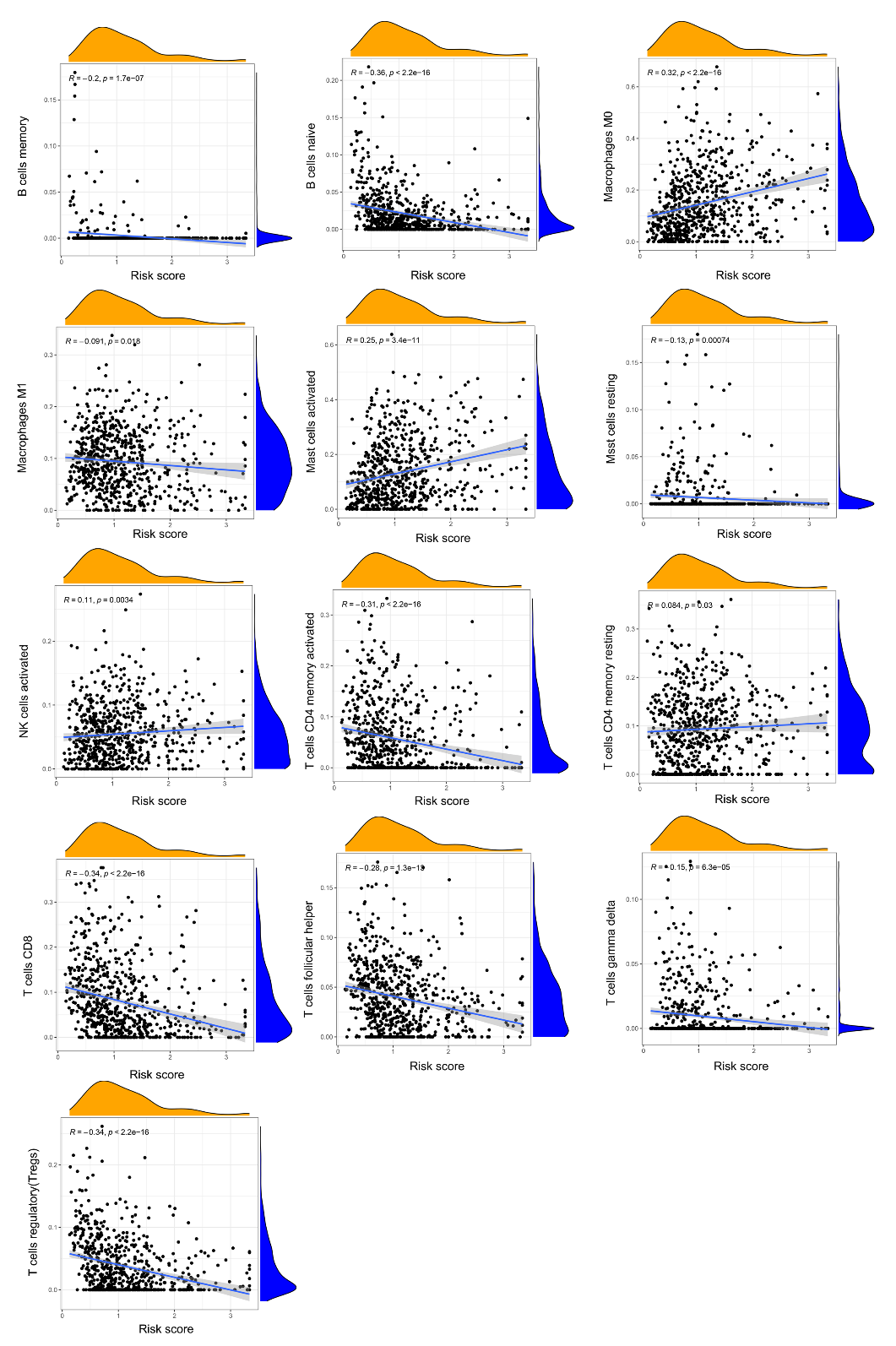


Figure S5 Correlation between risk scores and infiltrated immune cells.


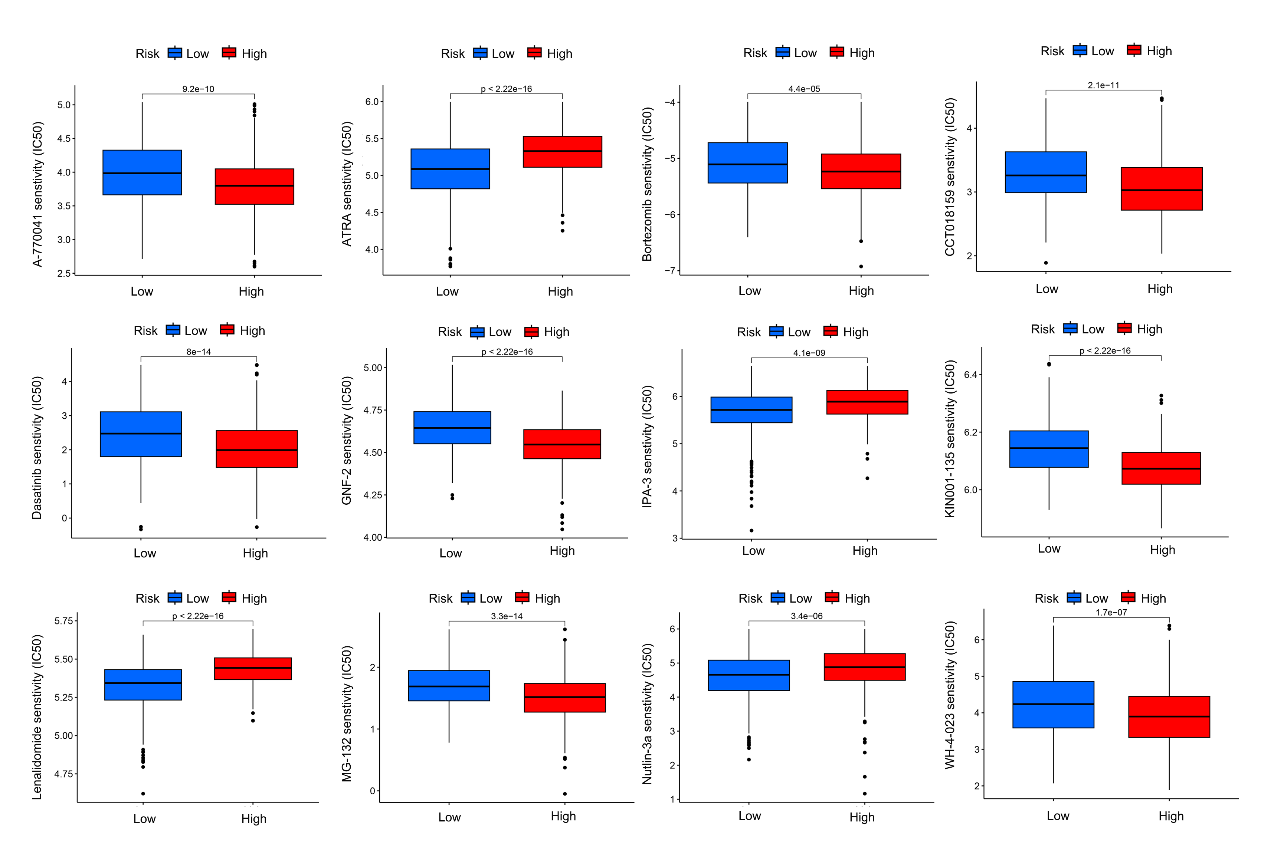


Figure S6 Drug sensitivity evaluation by the pRRophetic R package in high- and low-risk HNSCC patients.


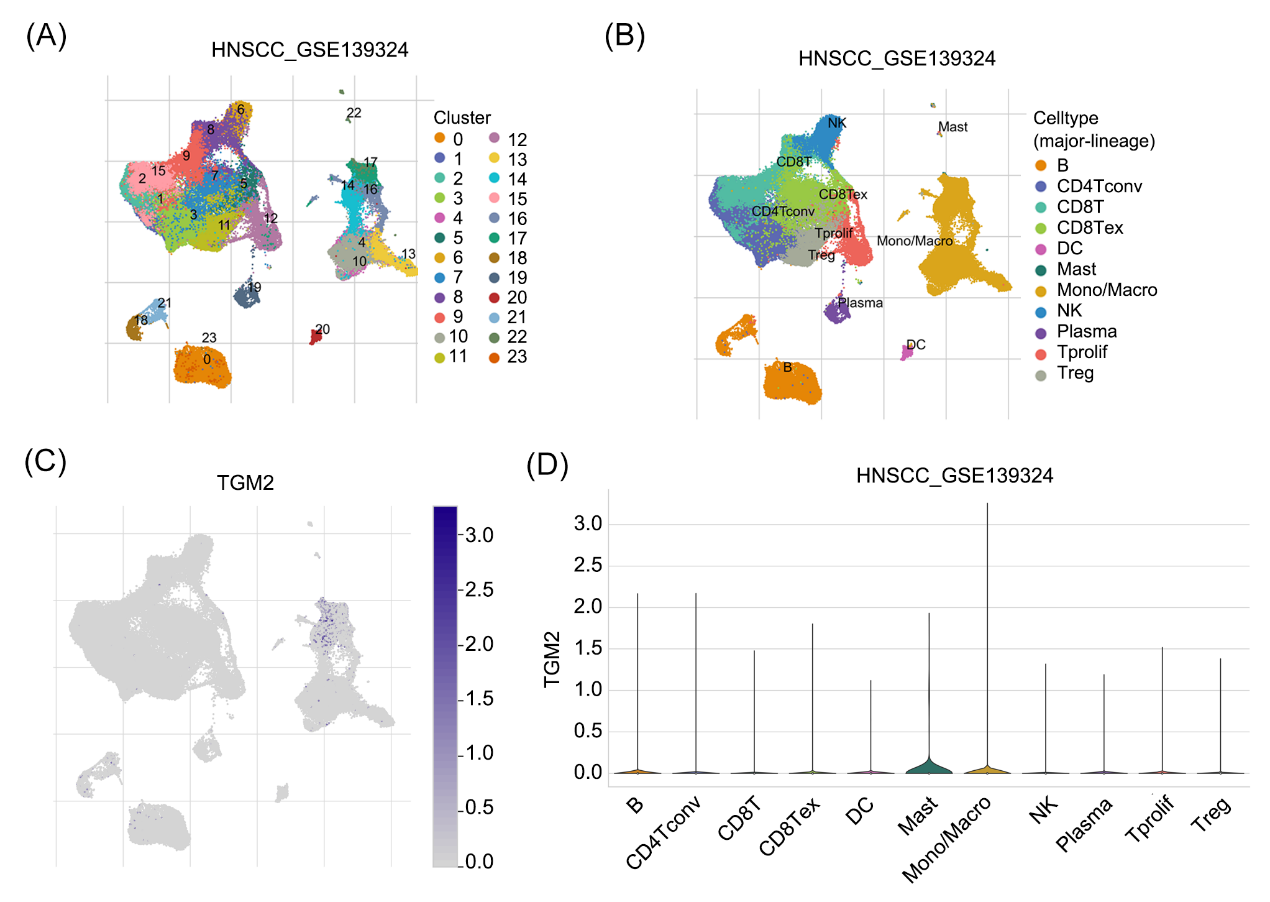


Figure S7 TGM2 expression analysis in single-cell RNA sequencing data. (A, B) Annotation of all cell types in GSE139324. (C-D) Expression of TGM2 in HNSCC tumor microenvironment (TME)-related cells.


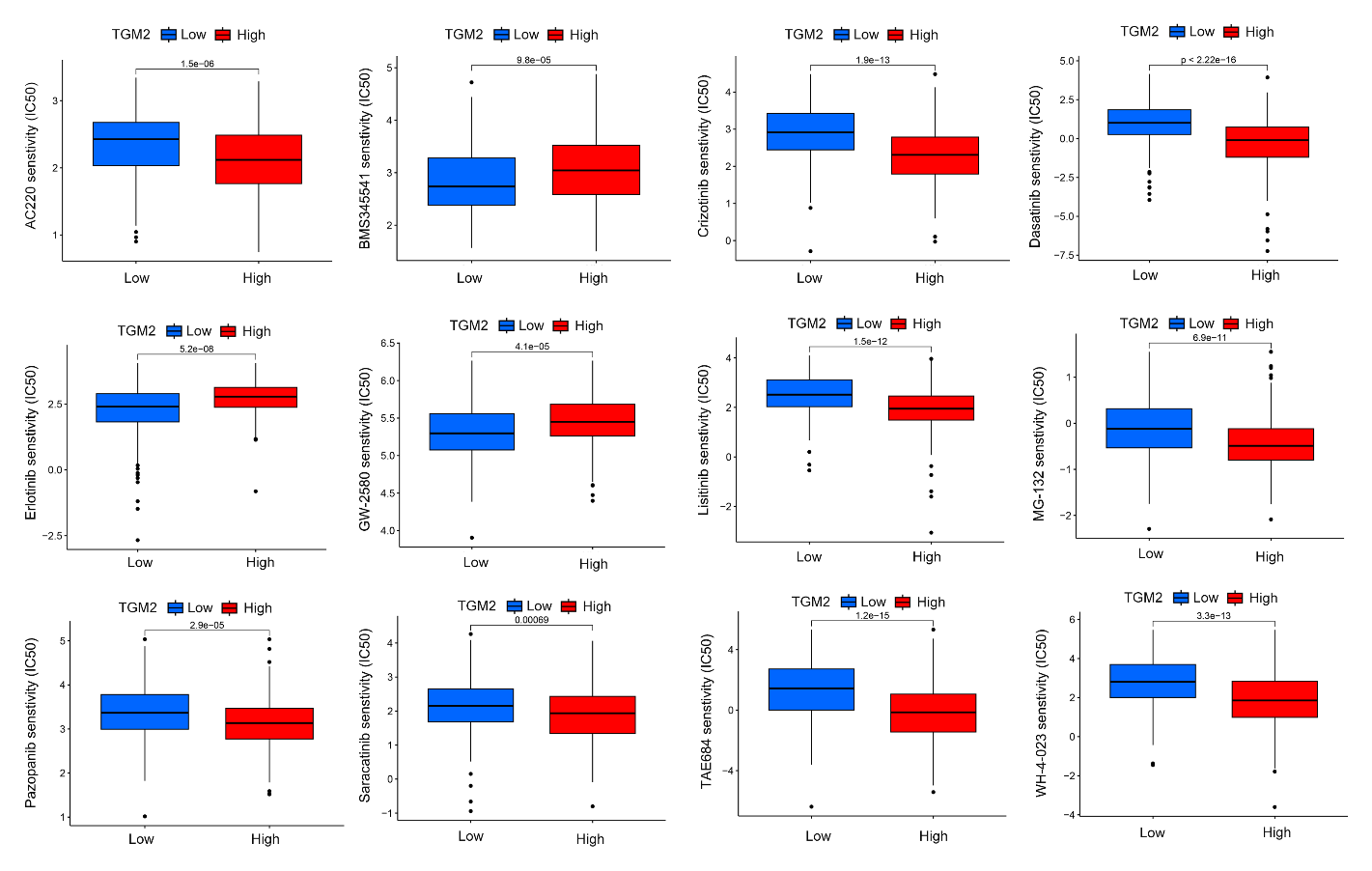


Figure S8 Drug sensitivity evaluation by the pRRophetic R package in HNSCC patients with high or low TGM2 expression.
